# Correlation analysis of serum reproductive hormones and metabolites during multiple ovulation in sheep

**DOI:** 10.1186/s12917-022-03387-1

**Published:** 2022-07-26

**Authors:** Quanzhong Xu, Chunwei Wang, Lequn Wang, Rui Feng, Yulin Guo, Shuang Feng, Liguo Zhang, Zhong Zheng, Guanghua Su, Lifen Fan, Chao Bian, Li Zhang, Xiaohu Su

**Affiliations:** 1 State Key Laboratory of Reproductive Regulation and Breeding of Grassland Livestock, Inner Mongolia University, No.49, Xilinguolenan Road, Hohhot, Inner Mongolia Autonomous Region 010017 People’s Republic of China; 2 School of Life Sciences, Inner Mongolia University, No.49, Xilinguolenan Road, Hohhot, Inner Mongolia Autonomous Region 010017 People’s Republic of China; 3 Ulanqab Agriculture and Animal Husbandry Bureau, Ulanqab Animal Husbandry Workstation, Ulanqab, Inner Mongolia Autonomous Region 012000 People’s Republic of China; 4 Department of Orthopedics, Ordos Central Hospital, Ordos, Inner Mongolia Autonomous Region 017000 People’s Republic of China; 5 Tumor Radiotherapy Department, Inner Mongolia People’s Hospital, Hohhot, Inner Mongolia Autonomous Region 010017 People’s Republic of China

**Keywords:** Multiple Ovulation, Blood reproductive hormone, Blood metabolome, Sheep

## Abstract

**Background:**

The establishment of non-invasive diagnostic method for multiple ovulation prediction is helpful to improve the efficiency of multiple ovulation. The blood hormones and metabolites would be suitable indexes for this subject.

**Methods:**

In this study, 86 estrus ewes (65 of induced estrus (IE) and 21 of spontaneous estrus (SE)) were selected and the blood samples were collected at the day before follicle-stimulating hormone (FSH) injection (1^st^) and before artificial insemination (2^nd^). The serum reproductive hormones ofFSH, luteinizing hormone (LH), 17β-Estradiol (E2), progesterone (P4) and anti-Mullerian hormone (AMH) were measured through enzyme linked immunosorbent assay (ELISA) and the untargeted metabolomics analysis was processed through LC–MS/MS. The embryos were collected after 6.5 days of artificial insemination.

**Results:**

In total, 975 and 406 embryos were collected in IE and SE group, respectively. The analysis of reproductive hormones showed that concentrations of FSH, E2 and AMH were positive correlated with the embryo yield while concentrations of LH and P4 were negative correlated in both group at 1^st^ detection. At 2^nd^ detection, the trends of reproductive hormones were similar with 1^st^ except P4, which was positive correlated with embryo yield. The metabolomics analysis showed that 1158 metabolites (721 in positive iron mode and 437 in negative iron mode) were detected and 617 were annotated. In 1^st^ comparation of high and low embryonic yield populations, 56 and 53 differential metabolites were identified in IE and SE group, respectively. The phosphatidyl choline (PC) (19:0/20:5) and PC (18:2/18:3) were shared in two groups. In 2^nd^ comparation, 48 and 49 differential metabolites were identified in IE and SE group, respectively. The PC (18:1/18:2) and pentadecanoic acid were shared. Most differential metabolites were significantly enriched in amino acid, fatty acid metabolism, digestive system secretion and ovarian steroidogenesis pathways.

**Conclusions:**

This study showed that FSH, P4, AMH, the PC relevant metabolites and some anomic acids could be potential biomarkers for embryonic yield prediction in ovine multiple ovulation. The results would help to explain the relation between blood material and ovarian function and provide a theoretical basis for the multiple ovulation prediction.

**Supplementary Information:**

The online version contains supplementary material available at 10.1186/s12917-022-03387-1.

## Summary sentence

This study showed that FSH, P4, AMH, the phosphatidyl choline (PC) relevant metabolites and some anomic acids could be potential biomarkers for embryonic yield prediction in ovine multiple ovulation.

## Background

The multiple ovulation and embryo transfer (MOET) technologies are applied to increase the progeny of excellent livestock. The donor can acquire tens of embryos at one estrus cycle through follicle-stimulating hormone (FSH) and other reproductive hormones stimulant. The MOET technologies were widely adopted within the sheep breeding industry since 1990s [1]. However, there still are some limiting factors affect the ovarian response during MOET processes, such as source and purity of hormones, administration protocols, breed, age, nutritional and reproductive status [2]. Even similar status of donor with the same multiple ovulation process, the outcomes vary widely. It would cause appreciable waste of drugs and donors. Although the ovary observation through ultrasonic detection could predict the outcome of embryo yield [3], it is a tedious work and bring the stress to donors.

The ovarian response is a complicated physiological process during multiple ovulation. The FSH, luteinizing hormone (LH), 17β-Estradiol (E2), progesterone (P4), anti-Mullerian hormone (AMH) and other reproductive hormones play roles together. The FSH and LH work together to promote follicle maturation, E2 secretion, ovulation, luteal production and maintenance [4]. E2 is secreted by granulosa cells and stimulates follicle development to induce estrus behavior, while low concentration of E2 can inhibit the secretion of FSH and LH [5]. P4 is one of main progesterone which is secreted by ovary and promotes the uterus receptivity to regulate the negative feedback of gonadotropin [6]. AMH is a product of small antral follicles and serves to function as an autocrine and paracrine regulator of follicular maturation [7]. In addition, AMH is clinically useful as a screening tool for diminished ovarian reserve [8]. Thus, the concentrations of reproductive hormones may be suitable parameters to predict the embryonic outcome during multiple ovulation.

Except hormones, the metabolites in blood also play an important role at ovarian response [9]. The metabolites (**molecular weight** < 1000) act as substrates and products in various metabolic pathways. The metabolome which collects the small-molecule chemical entities, has been studied and aimed to biomarkers identification in disease diagnosis and prediction. However, the value of metabolomics has been expended from a simple biomarker to a technology for discovering active drivers of biological processes [10]. Various techniques are used for metabolomics processing, include high performance liquid chromatography–mass spectrometry (LC–MS), chromatography–mass spectrometry (GC–MS), nuclear magnetic resonance (NMR) and so on [11]. Among these techniques, the Ultra-Performance LC–MS (UPLC–MS) has the characteristics of high-through put processing, high resolution, and high sensitivity to obtain more accurate, and comprehensive data [12]. Until now, there is few blood metabolome study for multiple ovulation, in which biomarkers may be found for embryonic yield.

Here, the Dairy Meade (DM) ewes with similar age and body weight were selected as donors. The blood samples were collected at the day before FSH injection and before artificial insemination (AI). The FSH, LH, E2, P4 and AMH were measured through enzyme linked immunosorbent assay (ELISA) and the untargeted metabolomics analysis was processed through LC–MS/MS. We aim to explain the relation of blood materials and ovarian response during multiple ovulation and provide a theoretical basis for the ovine multiple ovulation prediction.

## Methods

### Ethics Statement

This study received approval from Inner Mongolia University Research Ethics Committee (approval number: 2021002). All experiments were performed according to Chinese laws and institutional guidelines.

### Experimental location and ewes’ management

This study was conducted at Fengdongzhiying Husbandry Technology Co., Ltd. and Monterra Husbandry Technology Development Co., Ltd. in Ulanqab (Inner Mongolia, China) at 39°37′ ~ 43°28′ north latitude and 109°16′ ~ 114°49′ east longitude, altitude ranging from 1595 to 2150 m above sea level. This region has a tropical mid-temperate semi-arid continental monsoon climate, with long dry winters and short rainy summers.

The DM sheep is a type of New Zealand dairy sheep breed and is known for its high milk yield [13]. 86 sexually mature and clinically healthy DM ewes between 1 to 3 years old and with a body weight of 67.5 ± 2.5 kg were used in this study. The body type was well-proportioned. They were from Fengdongzhiying Husbandry Technology Co., Ltd. (55 ewes) and Monterra Husbandry Technology Development Co., Ltd (31 ewes). Design diet for ewes was designed, based on the nutritional needs, and unified management at barn feeding. Ewes were fed a total mixed ration consisting of 13.60% alfalfa Hay, 27.30% Leymus chinensis, 4.60% corn grass, 15.20% whole corn silage, 15.20% corn grain, 24.10% ewe concentrate supplement, and characterized with a forage concentrate ratio of 70:30.

### Multiple ovulation protocols

The multiple ovulation of DM ewes was processed at September to October of 2021.

At the start of experiment, the estrus was detected and ewes were divided into controlled internal drug release (CIDR) (Zoetis, New Zealand) induced estrus (IE) and Spontaneous estrus (SE) group (65 and 21, respectively).

**Fig. 1:**
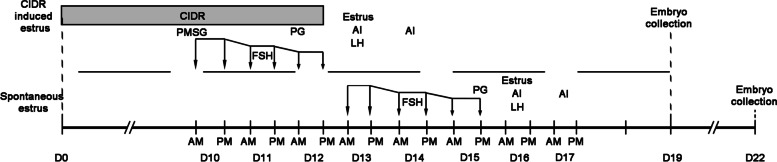
Schedule of ovine multiple ovulation. At the start of experiment, the estrus was detected and the ewes were divided into CIDR induced estrus (IE) and Spontaneous estrus (SE) group (65 and 21, respectively). The IE group was treated as upper: the CIDR was inserted into vagina of non-estrus ewes at random day (D0); at D10 to 12, the FSH was injected for 6 times; at first time of FSH injection, the PMSG was injected synchronously; at fifth time of FSH injection, the PG was injected synchronously; the CIDR was removed at last time of FSH injection; at D13, the estrus was detected and AI was performed twice (12 h interval); the LH was injected at first AI; at D19, the embryos were collected via surgical uterus flushing. The SE group was treated as under: the estrus day of ewes as D0; at D13 to 15, the FSH was injected for 6 times; at first time of FSH injection, at last time of FSH injection, the PG was injected synchronously; at D16, the estrus was detected and AI was performed twice (12 h interval); the LH was injected at first AI; at D22, the embryos were collected via surgical uterus flushing

The IE group was treated as follow: the CIDR was inserted into vagina of non-estrus ewes at random day (D0); at D10 to 12, a total of 250 μg FSH (Stimufol®, Belgium) was injected with 6 times at 10% decreasing doses, eg. the dose of 90% of first time was the dose of second time and the dose of 90% of second time was the dose of third time and so on; at first time of FSH injection, 250 IU of pregnant mare serum gonadotropin (PMSG) (Reprobiol, New Zealand) was injected synchronously; at fifth time of FSH injection, 150 μg of PG Cloprostenol (PG) (Reprobiol, New Zealand) was injected synchronously; the CIDR was removed at last time of FSH injection; at D13, the estrus was detected and AI was performed twice (12 h interval); 100 IU of LH (Reprobiol, New Zealand) was injected at first AI; at D19, the embryos were collected via surgical uterus flushing after anesthetized with lidocaine hydrochloride (Fig. 1).

The SE group was treated as follow: the estrus day of ewes as D0; at D13 to 15, a total of 250 μg FSH was injected with 6 times at 10% decreasing doses; at first time of FSH injection, at last time of FSH injection, 150 μg of PG was injected synchronously; at D16, the estrus was detected and AI was performed twice (12 h interval); 100 IU of LH (Reprobiol, New Zealand) was injected at first AI; at D22, the embryos were collected via surgical uterus flushing after anesthetized with lidocaine hydrochloride (Fig. 1).

### Embryo collection

The embryo collection protocol was followed as Bergstein-Galan et al. [14]. Brifely, donors were sedated with acepromazine 1% (0.05 mg/kg, IM) and inhalation anaesthesia induced with isoflurane. A skin incision approximately 3 cm in length was continued through subcutaneous tissue, abdominal musculature and peritoneum on the alba line. Then, uterine horns were external‐ized. A Foley catheter was positioned in the proximal third of each uterine horn. After the inflation of the probe balloon, the surgeon inserted a 20G catheter into the distal third of the uterine horn. 30 millilitres of PBS was infused from the catheter and collected through the Foley probe into a Petri dish in each uterine horn. Finally, uterine horns were repositioned and the musculature, subcutaneous and skin were sutured.

### Blood sampling

All ewes of blood samples were collected at mornings of D10, D13 for IE group and D13, D16 for SE group, the time before FSH injection or AI. Blood samples were collected in vacuum blood collection tubes. Then, the blood was centrifuged at 10,000 × g for 10 min to obtain the serum. All serum samples were stored in liquid nitrogen until further use.

### Reproductive hormonal analyses

Followed total embryonic yield ≥ 20 as high embryonic yield (HEY) and ≤ 14 as low embryonic yield (LEY) population, five ewes of each populations (*n* = 5) were selected for serum reproductive hormones analysis.

The concentrations of serum reproductive hormones (FSH, LH, E2, P4 and AMH) were determined by ELISA kits (Sangon Biotech, Shanghai, China) according to the manufacturer’s instructions. The ELISA plates were read with a microplate reader (Thermo Scientific, Shanghai, China) to record the optical densities. Each standard curve was drew and the concentration of each sample was calculated.

### LC–MS/MS measurements for serum metabolomics

Ten ewes of HEY and LEY populations of IE group (*n* = 10) and six ewes of HEY and LEY populations of SE group (*n* = 6) were selected for serum metabolomics analysis.

The LC–MS/MS based serum metabolomics analysis was processed by Novogene Co. LTD. (Beijing, China).

Sample preparation was performed according to a previous report with slight modification [15]. Briefly, the serums (100 μL) were placed in the epoxy resin **(**EP) tubes and resuspended with prechilled 80% methanol by well vortex. Then the samples were incubated on ice for 5 min and centrifuged at 15,000 g, 4 °C for 20 min. Some of supernatant was diluted to final concentration containing 53% methanol by LC–MS grade water. The samples were subsequently transferred to a fresh EP tube and then were centrifuged at 15,000 g, 4 °C for 20 min. Finally, the supernatant was injected into the LC–MS/MS system analysis.

UHPLC-MS/MS analyses were performed using a Vanquish UHPLC system (Thermo Fisher, Germany) coupled with an Orbitrap Q ExactiveTM HF mass spectrometer (Thermo Fisher, Germany). Samples were injected onto a Hypesil Goldcolumn (100 × 2.1 mm, 1.9 μm) using a 17-min linear gradient at a flow rate of 0.2 mL/min. The eluents for the positive polarity mode were eluent A (0.1% FA in Water) and eluent B (Methanol) and the negative polarity mode were eluent A (5 mM ammonium acetate, pH 9.0) and eluent B (Methanol). The solvent gradient was set as follows: 2% B, 1.5 min; 2–85% B, 3 min; 100% B, 10 min; 100–2% B, 10.1 min; 2% B, 12 min. Q ExactiveTM HF mass spectrometer was operated in positive/negative polarity mode with spray voltage of 3.5 kV, capillary temperature of 320 °C, sheath gas flow rate of 35 psi and aux gas flow rate of 10 L/min, S-lens RF level of 60, Aux gas heater temperature of 350 °C.

### Data processing and metabolite identification

The data processing and metabolite identification were performed according to a previous report with slight modification [11]. Briefly, raw data files generated by UHPLC-MS/MS were processed using the Compound Discoverer 3.1 (CD3.1, Thermo Fisher) to perform peak alignment, peak picking and quantitation for each metabolite. The normalized data was used to predict the molecular formula based on additive ions, molecular ion peaks and fragment ions. And then peaks were matched with the mzCloud (https://www.mzcloud.org/), mzVault and MassList database to obtain the accurate qualitative and relative quantitative results.

### Data analysis

The metabolomics statistical analyses were performed using statistical software R (R version R-3.4.3), Python (Python 2.7.6 version) and CentOS (CentOS release 6.6). The normal transformations were attempted using of area normalization method if the data was not normally distributed. The Kyoto Encyclopedia of Genes and Genomes (KEGG, http://www.kegg.com), LIPID MAPS (Lipidmaps, www.lipidmaps.org) and Human Metabolome Database (HMDB, http://www.hmdb.ca) were used to annotate the biological functions of metabolites. Principal components analysis (PCA) and Partial least squares discriminant analysis (PLS-DA) were performed at metaX [16]. The univariate analysis (t-test) was applied to calculate the statistical significance (*P*-value).The metabolites with Variable Importance in Projection (VIP) > 1, *P*-value < 0.05 and Fold Change (FC) ≥ 1.2 or ≤ 0.83 were considered to be differential metabolites. Volcano plots were used to filter metabolites of interest which based on log_2_(Fold Change) and -log_10_(*P*-value) of metabolites by ggplot2 in R language. The functions of differential metabolites were studied using the KEGG database [17–19].

## Results

### Embryo production

In this study, 65 with CIDR induced estrus (IE) and 21 with spontaneous estrus (SE) ewes were selected and treated with standard multiple ovulation process (Fig. 1). The result was showed in Table 1: A total of 975 and 406 embryos (15.0 ± 8.1 and 19.3 ± 10.2 of average) were collected in IE and SE groups, respectively. 608 and 327 embryos (9.4 ± 8.6 and 15.6 ± 9.4 of average) were viable in IE and SE groups, respectively. 15 of the IE group and 7 of the SE group were HEY population, 35 of the IE group and 7 of the SE group were LEY population, respectively.

**Table 1:** Statistics of embryonic production after multiple ovulation of ewes (Mean ± S.D.)

Estrus mode	CIDR induced estrus	Spontaneous estrus
Dnonr number	65	21
Total embryos	975	406
Average total embryos	15.0 ± 8.1	19.3 ± 10.2
Total viable embryos	608	327
Average viable embryos	9.4 ± 8.6	15.6 ± 9.4
High embryonic yield donor number (embryonic yield ≥ 20)	15 (23.08%)	7 (33.33%)
Average total embryos of high yield	26.3 ± 6.4	30.1 ± 9.1
Average viable embryos of high yield	17.0 ± 11.1	23.9 ± 10.4
Low embryonic yield donor number (embryonic yield ≤ 14)	35 (53.85%)	7 (33.33%)
Average total embryos of low yield	9.4 ± 3.7	10.7 ± 4.3
Average viable embryos of low yield	5.8 ± 5.1	8.3 ± 5.6

### Measurement of serum reproductive hormones

The result of serum reproductive hormones measurement was showed in Table 2. In 1^st^ measurement (the day of first FSH injection), the concentrations of FSH, E2 and AMH of HEY population were significantly higher than LEY population in both groups (*P* < 0.05); while the LH and P4 of HEY population were significantly lower than LEY population in both groups (*P* < 0.05). Specially, the largest variation between two populations is LH, followed by P4 and FSH.

**Table 2:** Analysis of serum reproductive hormones after multiple ovulation of ewes (*n* = 5. Mean ± S.D.)

Estrus mode	Hormone	1^st^		2^nd^	
		High yield	Low yield	High yield	Low yield
CIDR induced estrus	FSH (mIU/mL)	18.62 ± 0.41^a^	16.06 ± 0.12^b^	19.29 ± 0.25^a^	18.10 ± 0.15^b^
	LH (mIU/mL)	36.20 ± 0.72^a^	46.96 ± 0.78^b^	49.14 ± 0.59^a^	53.78 ± 1.15^b^
	E2 (pg/mL)	236.39 ± 6.41^a^	212.23 ± 2.82^b^	273.09 ± 5.49^a^	228.05 ± 7.01^b^
	P4 (ng/mL)	40.68 ± 2.44^a^	48.04 ± 0.69^b^	68.64 ± 4.54^a^	58.51 ± 3.70^b^
	AMH (ng/mL)	186.50 ± 2.86^a^	172.37 ± 1.44^b^	242.08 ± 6.25^a^	179.51 ± 5.69^b^
Spontaneous estrus	FSH (mIU/mL)	19.08 ± 0.41^a^	16.58 ± 0.17^b^	19.56 ± 0.31^a^	18.25 ± 0.26^b^
	LH (mIU/mL)	34.43 ± 0.62^a^	47.36 ± 0.51^b^	47.83 ± 0.61^a^	52.46 ± 1.03^b^
	E2 (pg/mL)	241.03 ± 7.28^a^	216.32 ± 3.32^b^	278.94 ± 7.38^a^	223.42 ± 6.76^b^
	P4 (ng/mL)	38.72 ± 3.03^a^	44.65 ± 0.59^b^	69.46 ± 5.53^a^	57.32 ± 3.85^b^
	AMH (ng/mL)	187.24 ± 3.61^a^	174.73 ± 1.48^b^	237.85 ± 5.96^a^	181.45 ± 6.27^b^

^a,^^b^Means significant difference between two polulations of same time and group (*P* < 0.05)

At 2^nd^ measurement (the day before AI), the FSH, E2, P4 and AMH of HEY population were significantly higher than LEY population in both groups (*P* < 0.05); while the LH was significantly lower in HEY population than LEY population in both groups (*P* < 0.05). Specially, the largest variation between two populations is AMH, followed by E2 and P4.

### Metabolomics analysis of serum

Untargeted metabolic profiling of ewes’ serum at multiple ovulation.

To explore the serum metabolic differences between HEY and LEY populations of each group, the untargeted metabonomics analysis was performed. In total, 350 annotated metabolites from 721 positive-ion features (Table S 1) and 267 annotated metabolites from 437 negative-ion features were identified (Table S 2).

### Multivariate statistical analysis

#### Multivariate statistical analysis of 1^st^ sampling

**Fig. 2:**
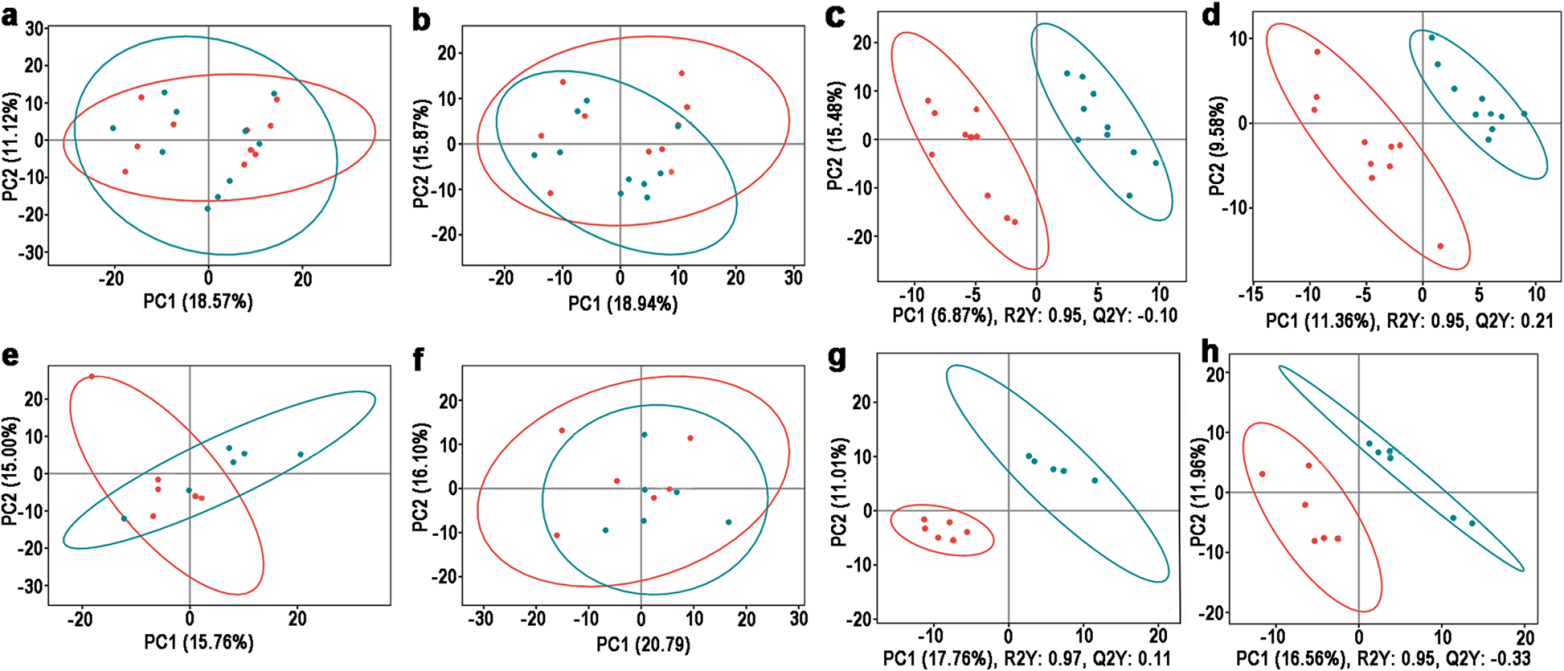
The first time (1^st^) Metabolomic analysis of serum from ewes with multiple ovulation treatment. PCA analysis results of IE group under positive ion mode (**a**) and negative ion mode (**b**) and SE group under positive ion mode (**c**) and negative ion mode (**d**). PLS-DA analysis results of IE group under positive ion mode (**c**) and negative ion mode (**d**) and SE group under positive ion mode (**g**) and negative ion mode (**h**)

The PCA was used to determine the sample separation and aggregation between HEY and LEY populations. In IE group of positive-ion mode, the PCA scores illustrated that PC1 and PC2 were 18.57% and 11.12% of the variation, respectively (Fig. 2a); PC1 and PC2 were 18.94% and 15.87% of the variation in negative-ion mode, respectively (Fig. 2b). In SE group, PC1 and PC2 were 15.76% and 15.00% of the variation in positive-ion mode, respectively (Fig. 2c); PC1 and PC2 were 20.79% and 16.10% of the variation in negative-ion mode, respectively (Fig. 2d).

The PLS-DA was used to better understand the variables responsible for the classification and achieve a higher level of group separation. In IE group of positive-ion mode, the R2 of the PLS-DA model was 0.95 and the Q2 was—0.10 (Fig. 2e); R2 and Q2 were 0.95 and 0.21 in negative-ion mode, respectively (Fig. 2f). In SE group, R2 and Q2 were 0.97 and 0.11 in positive-ion mode (Fig. 2g); R2 and Q2 were 0.95 and -0.33 in negative-ion mode (Fig. 2h), respectively.

#### Multivariate statistical analysis of 2^nd^ sampling

**Fig. 3:**
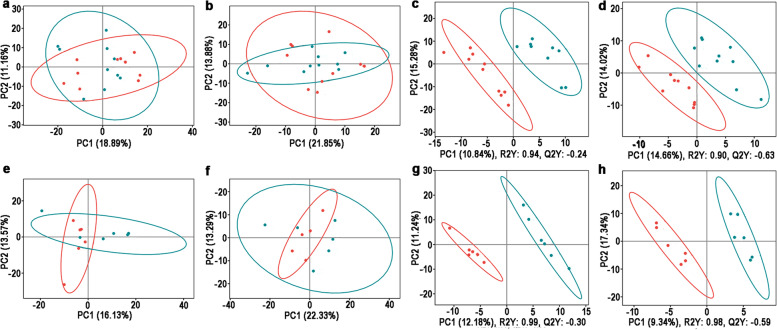
The second time (2^nd^) Metabolomic analysis of serum from ewes with multiple ovulation treatment. PCA analysis results of IE group under positive ion mode (**a**) and negative ion mode (**b**) and SE group under positive ion mode (**c**) and negative ion mode (**d**). PLS-DA analysis results of IE group under positive ion mode (**c**) and negative ion mode (**d**) and SE group under positive ion mode (**g**) and negative ion mode (**h**)

In IE group of positive-ion mode, the PCA scores of PC1 and PC2 were 18.89% and 11.16% of the variation, respectively (Fig. 3a); PC1 and PC2 were 21.85% and 13.88% of the variation in negative-ion mode, respectively (Fig. 3b). In SE group, PC1 and PC2 were 16.13% and 13.57% of the variation in positive-ion mode, respectively (Fig. 3c); PC1 and PC2 were 22.33% and 13.29% of the variation in negative-ion mode, respectively (Fig. 3d).

In IE group of positive-ion mode, the R2 of the PLS-DA model was 0.94 and the Q2 was -0.24 (Fig. 3e); R2 and Q2 were 0.90 and -0.63 in negative-ion mode, respectively (Fig. 3f). In SE group, R2 and Q2 were 0.99 and -0.30 in positive-ion mode (Fig. 3g); R2 and Q2 were 0.98 and -0.59 in negative-ion mode (Fig. 3h), respectively.

### Identification of differential metabolites and functional enrichment

The LC–MS data was used to analyze the metabolites of different substances. The differential metabolites were screened based on the following criteria: VIP ≥ 1; FC > 1.2 or < 0.83; *P* ≤ 0.05, a comprehensive statistical analysis was performed.

#### Identification of differential metabolites of 1^st^ sampling

**Fig. 4:**
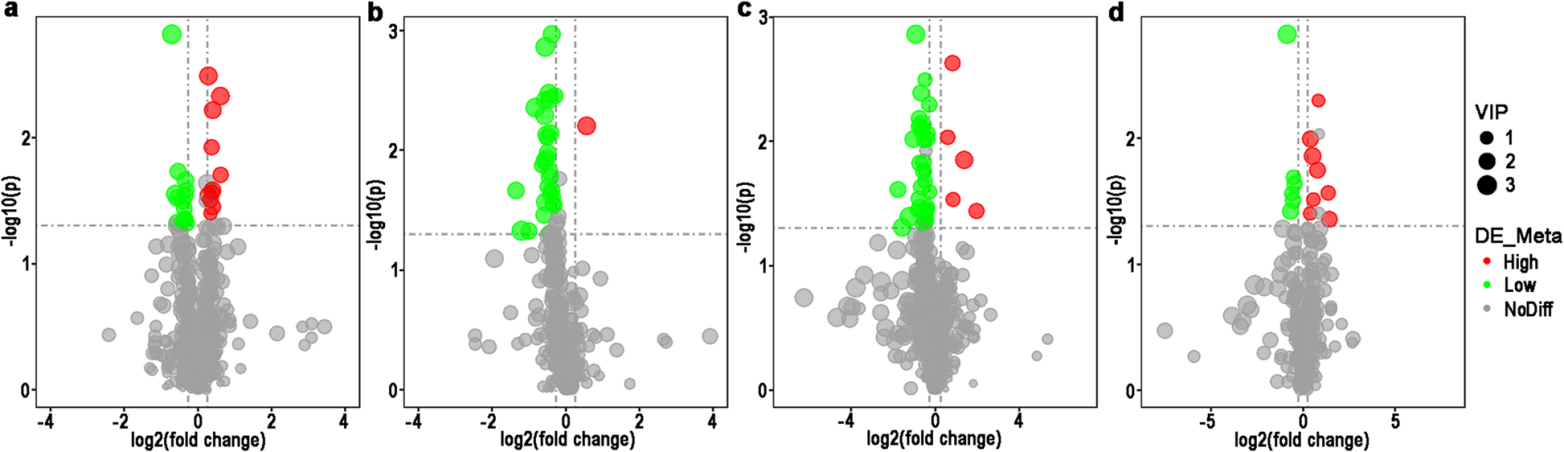
Volcano plot of first comparation analysis (1^st^) between high and low embryonic yield populations of serum from ewes with multiple ovulation treatment. IE group under positive ion mode (**a**) and negative ion mode (**b**) and SE group under positive ion mode (**c**) and negative ion mode (**d**)

In IE group, 26 and 30 differential metabolites were identified at positive-ion and negative-ion modes, respectively (Fig. 4a and b, Table S 3). In SE group, 39 and 14 differential metabolites were identified at positive-ion and negative-ion modes, respectively (Fig. 4c and d, Table S 4). Especially, the PC (19:0/20:5) and PC (18:2/18:3) were shared significant metabolites in two groups (Table 3).

**Table 3:** Shared metabolites of IE and SE groups at 1^st^ comparation

Metabolite	Molecular formula	Estrus mode	Fold change^a^	*P* value^b^	VIP^c^	Up.Down^d^
phosphatidyl choline (19:0/20:5)	C47 H84 N O8 P	CIDR induced estrus	0.65	0.0281	2.85	down
		Spontaneous estrus	0.65	0.0458	1.52	down
phosphatidyl choline (18:2/18:3)	C44 H78 N O8 P	CIDR induced estrus	0.78	0.0367	2.03	down
		Spontaneous estrus	0.74	0.0398	1.51	down

^a^The fold change of the high embryonic yield population vs low embryonic yield population (a higher ratio indicates a higher level of expression of a compound in the high embryonic yield population)

^b^*P* value is the significance level of the difference between two populations

^c^Variable Importance in the Projection of two populations

^d^Compared with the low embryonic yield population, the high embryonic yield population presents up or down expressed of this metabolite

#### Identification of differential different metabolites of 2^nd^ sampling

**Fig. 5:**
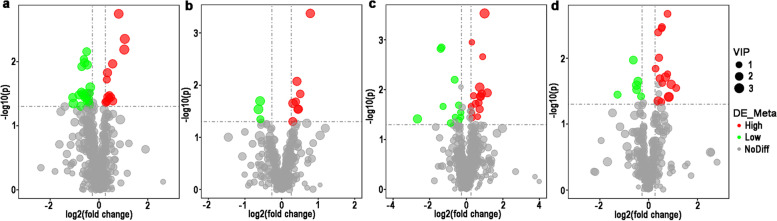
Volcano plot of second comparation analysis (2^nd^) between high and low embryonic yield populations of serum from ewes with multiple ovulation treatment. IE group of positive ion mode (**a**) and negative ion mode (**b**) and SE group of positive ion mode (**c**) and negative ion mode (**d**)

In IE group, 37 and 11 differential metabolites were identified at positive-ion and negative-ion modes, respectively (Fig. 5a and b, Table S 5). In SE group, 27 and 22 differential metabolites were identified at positive-ion and negative-ion modes, respectively (Fig. 5c and d, Table S 6). Especially, the phosphatidyl choline (PC) (18:1/18:2) and pentadecanoic acid were shared differential metabolites in two groups (Table 4).

**Table 4:** Shared metabolites of IE and SE groups at 2^nd^ comparation

Metabolite	Molecular formula	Estrus mode	Fold change^a^	*P* value^b^	VIP^c^	Up.Down^d^
PC (18:1/18:2)	C44 H82 N O8 P	CIDR induced estrus	0.75	0.0383	1.61	down
		Spontaneous estrus	1.24	0.0011	1.01	up
Pentadecanoic acid	C15 H30 O2	CIDR induced estrus	0.67	0.0451	1.39	down
		Spontaneous estrus	1.47	0.0238	1.34	up

^a^The fold change of the high embryonic yield population vs low embryonic yield population (a higher ratio indicates a higher level of expression of a compound in the high embryonic yield population)

^b^*P* value is the significance level of the difference between two populations

^c^Variable Importance in the Projection of the two populations

^d^Compared with the low embryonic yield population, the high embryonic yield population presents up or down expressed of this metabolite

### Functional enrichment of differential metabolites

The functional enrichment of differential metabolites was followed as Kanehisa et al. [17–19]. In 1^st^ analysis, the KEGG pathway enrichment analysis showed that 27 metabolic pathways were enriched (18 at positive-ion and 9 at negative-ion mode) in IE group (Fig. S 1a and S 1b). 18 metabolic pathways were enriched (15 at positive-ion and 3 at negative-ion mode) in SE group (Fig. S 1c and S 1d). The enriched pathways include glycerophospholipid metabolism, amino acid metabolic, fatty acid metabolism, digestive system secretion and so on.

In 2^nd^ analysis, 20 metabolic pathways were enriched (7 at positive-ion and 13 at negative-ion mode) in IE group (Fig. S 2a and S 2b). 24 metabolic pathways were enriched (19 at positive-ion and 5 at negative-ion mode) s in SE group (Fig. S 2c and S 2d). The enriched pathways include amino acid metabolic, steroidogenesis, fatty acid metabolism pathways and so on.

## Discussion

The ovarian response during multiple ovulation is regulated by many factors. In addition, some substances which are secreted by ovary would affect the physiological process of donor. Here, we found that concentrations of FSH, E2 and AMH were positive correlated with the embryo yield while the level of LH and P4 were negative correlated before exogenous FSH injection. After follicular development, the correlations were similar with before except P4, which was positive correlated with embryonic yield. And most differential metabolites include PC relevant metabolites and some anomic acids were enriched at glycerophospholipid metabolism, amino acid metabolic, fatty acid metabolism, digestive system secretion pathways.

Previous study showed that high concentration of FSH can improve the recruitment of primordial follicles, stimulate the development of antral follicles and granulose cells, then generate follicle-stimulating receptors [20]. In this study, higher concentration of FSH achieve better embryonic yield, which may be a candidate indicator in ovine multiple ovulation. The increase of LH is necessary in ovulation. However, it is neither too high nor too low [21]. Here we found that the concentration of LH was negative correlated with the embryonic yield in both detection. However, the mechanism and special range are needed to further exploration. The concentration of E2 is relatively higher at the time of preceding ovulation [22]. However, previous study showed that an injection of E2 did not affect the embryonic yield of IE ewes during multiple ovulation [23, 24]. So, it may not suitable to be a candidate indicator in ovine multiple ovulation. Low concentration of P4 is necessary for maternal estrus. In Chios sheep, P4 was considered to be an indicator of ovarian response during multiple ovulation, in which donors with higher P4 at estrous achieve better ovarian response and embryonic yield [25]. Based on this result, we further found that lower concentration of P4 before FSH injection owned higher embryonic yield. In the process of multiple ovulation, the injection of exogenous FSH only is helpful to promote the development of small luminal follicles or rescues early atretic follicles, not recruit the preluminal follicles. The AMH is secreted by small luminal follicles and can promote their maturation. It was demonstrated that AMH is a predictor of fertility and response to multiple ovulation in cattle and sheep [26]. We further certified that AMH is predictive to ovarian response at earlier time of multiple ovulation in sheep. In total, the FSH, P4 and AMH would be suitable predictors for ovine multiple ovulation.

In this study, the PC relevant metabolites were found to be significantly different between HEY and LEY populations. PC is the most abundant phospholipid in mammalian cell membranes, which constitutes 40 ~ 50% of total phospholipids. Additionally, PC is an indispensable substance in cells, plays a key role in the transmission of cell signals. Research showed that different PCs in follicular fluid of bovine during multiple ovulation and estrous synchronization treatment appeared different variation, which indicated that ovarian superstimulation seems to modulate the phospholipid content of follicular fluid with a significant increase and would be a suitable biomarker involved with reproductive processes successful as multiple ovulation response and embryonic development [27]. The human ovarian stimulation study showed that some lipids in follicular fluid which were belonged to PC were present in higher concentrations in succeed group and may be useful as biomarkers for therapeutic intervention in women with poor ovarian response [28]. However, there is less report to demonstrate that PC relevant metabolites in serum could be biomarkers for multiple ovulation. Our study result showed that PC (19:0/20:5) and PC (18:2/18:3) may be serum predictors in ovine multiple ovulation.

The amino acids and peptides present important bioactivity in lots of biological processes. Serious studies demonstrated that the supplement of amino acids is necessary in the development of zygote, which now are known to be involved in intermediary metabolism, as energy substrates, in signal transduction, osmoregulation and as intermediaries in numerous pathways which involve nitrogen metabolism, e.g., the biosynthesis of purines, pyrimidines, creatine and glutathione. [29–32]. Although there are species differences, all amino acids are selectively transported into oviduct fluids at all reproductive stages. Therefore, some special amino acids may contribute to the embryonic development during multiple ovulation. In this study, the lysine, cystine, arginine and threonine were found to present higher concentration in HEY population of IE or SE groups before first FSH injection or AI. An porcine dietary lysine intake research showed that low lysine intake in sows impaired follicular development and reduced the ability of follicles to support oocyte maturation [33]. High lysine intake can increase the insulin and insulin-Like Growth Factor 1 (IGF-1), and the IGF-1 could promote the development and maturation of follicles during multiple ovulation in sows [34, 35]. It suggested that higher lysine intake could help to follicular development. In in vitro maturation and embryos culture of goat and cattle, the addition of cystine increases the GSH level and is helpful to overcome the oxidative stress and and blastulation [36–38]. Here, we demonstrated that higher concentration of cystine in serum is favorable for embryonic yield during multiple ovulation of ovine. Arginine is thought to have a pivotal role in regulating embryonic growth and differentiation, particularly at the blastocyst stage, which preferentially triggers the mTORC1 signalling complex [39]. Arginine is also the precursor for the free radical nitric oxide (NO) which is produced by the enzyme NO synthase. In mouse preimplantation embryo, NO limits oxygen consumption at the blastocyst stage [40]. It is inferred that higher arginine would helpful for the embryonic development in vivo. In oocyte, the serine/threonine protein kinase Akt participates in the control of meiosis resumption and, at metaphase II stage, regulates polar body emission and spindle organization. Its inhibition negatively affects preimplantation embryo development. The creatine and carnosine were also found to positive correlate with the embryonic yield. Both of them are stored in muscle and serve as energy and antioxidation activities. In total, the serum lysine, cystine, arginine, threonine, creatine and carnosine would be potential biomarkers for ovine multiple ovulation.

## Conclusion

The serum hormones and metabolome analysis provide a feasible approach to explore predictor of multiple ovulation. This study showed that FSH, P4, AMH, PC relevant metabolites and some anomic acids could be potential biomarkers for embryonic yield prediction in ovine multiple ovulation. The results would help to explain the relation between blood material and ovarian function and provide a theoretical basis for the multiple ovulation prediction. The accuracy of relevant factors needs to further investigation.

## Supplementary Information

**Additional file 1: Fig S1. **KEGG enrichment of 1^st^ significantly differential metabolites. IE group of positive ion mode (a) and negative ion mode (b) and SE group of positive ion mode (c) and negative ion mode (d). **Fig S2. **KEGG enrichment of 2^nd^ significantly differential metabolites. IE group of positive ion mode (a) and negative ion mode (b) and SE group of positive ion mode (c) and negative ion mode (d).

**Additional file 2: Table S1.** Identified metabolites of ovine serum during multiple ovulation at positive ion mode.

**Additional file 3: Table S2.** Identified metabolites of ovine serum during multiple ovulation at negative ion mode.

**Additional file 4: Table S3. **Differential metabolites of IE group at 1^st^ comparation.

**Additional file 5: Table S4.** Differential metabolites of SE group at 1^st^ comparation.

**Additional file 6: Table S5.** Differential metabolites of IE group at 2^nd^ comparation.

**Additional file 7: Table S6.** Differential metabolites of SE group at 2^nd^ comparation.

## Data Availability

The dataset is available from https://db.cngb.org/cnsa/metabolize/page/sub033281/view.
