# Supplementary material for: Correlation analysis of serum reproductive hormones and metabolites during multiple ovulation in sheep
Source: BMC Vet Res. 2022 Jul 26;18:290. doi: 10.1186/s12917-022-03387-1 (PMC9317590; doi:10.1186/s12917-022-03387-1)
Supplement: Supplementary file 1 — Additional file 1: Fig S1. KEGG enrichment of 1st significantly differential metabolites. IE group of positive ion mode (a) and negative ion mode (b) and SE group of positive ion mode (c) and negative ion mode (d). Fig S2. KEGG enrichment of 2nd significantly differential metabolites. IE group of positive ion mode (a) and negative ion mode (b) and SE group of positive ion mode (c) and negative ion mode (d). [file 12917_2022_3387_MOESM1_ESM.pdf]

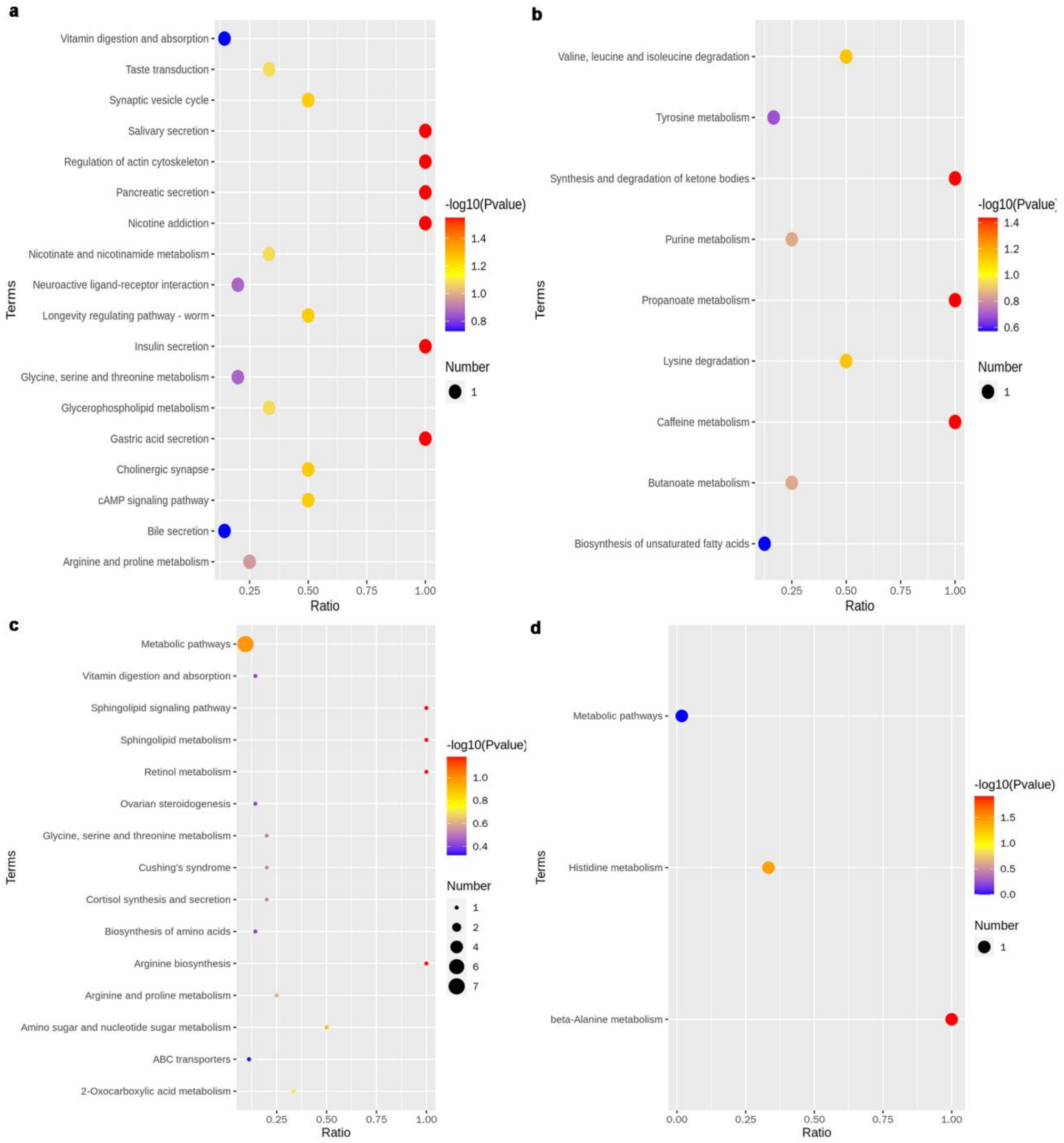

Fig S1 KEGG enrichment of 1st significantly differential metabolites.
IE group of positive ion mode (a) and negative ion mode (b) and SE group of positive ion mode (c) and negative ion mode (d).

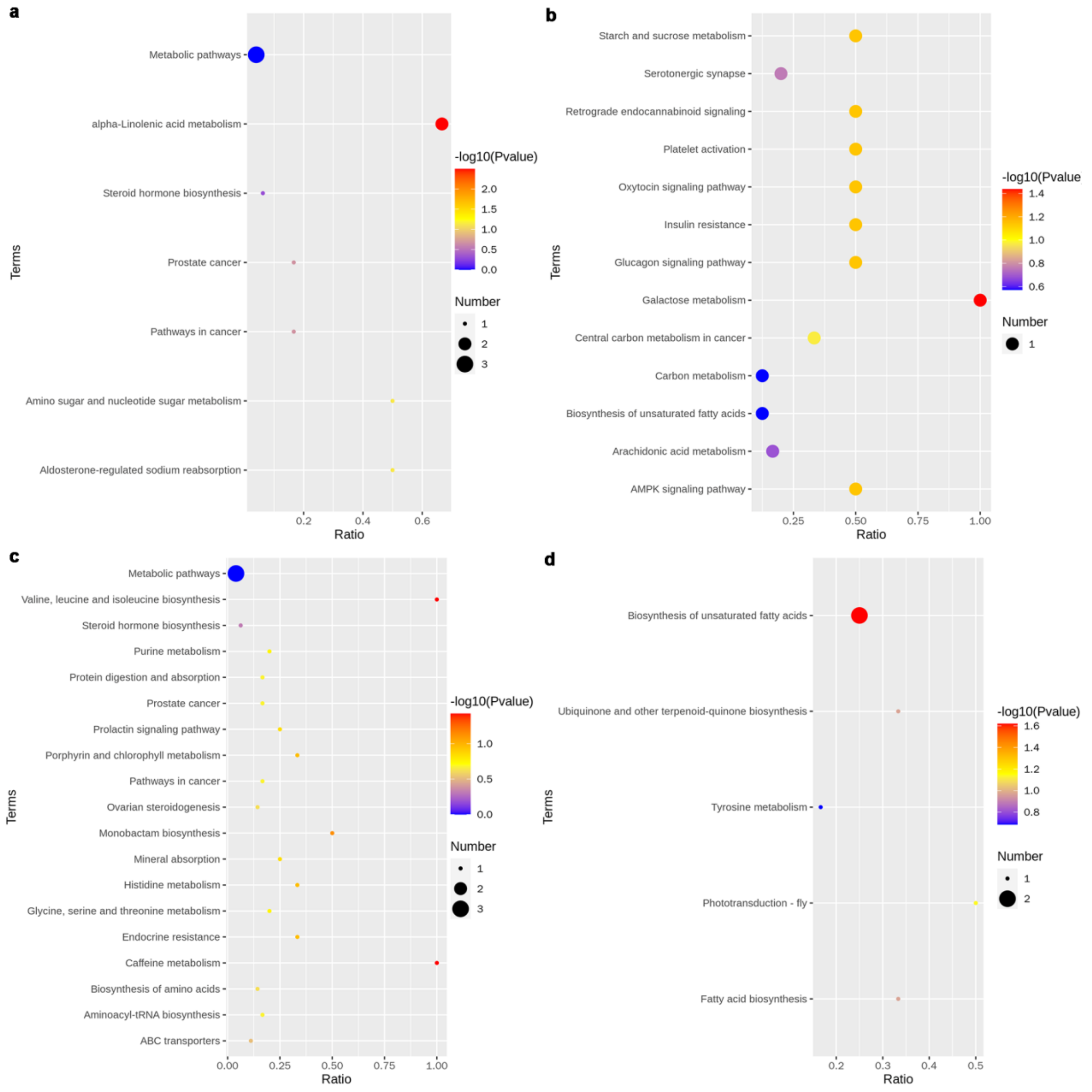

Fig S2 KEGG enrichment of 2nd significantly differential metabolites.

IE group of positive ion mode (a) and negative ion mode (b) and SE group of positive ion mode (c) and negative ion mode (d).
